# Effects of dietary fiber in gestating sow diets — A review

**DOI:** 10.5713/ab.23.0206

**Published:** 2023-08-28

**Authors:** Hyunwoong Jo, Beob Gyun Kim

**Affiliations:** 1 Department of Animal Science and Technology, Konkuk University, Seoul 05029, Korea

**Keywords:** Dietary fiber, Microbiota, Reproductive Performance, Sows

## Abstract

The objective of this review was to provide an overview of the effects of dietary fiber (DF) on reproductive performance in gestating sows. Dietary fibers have been suggested to modulate microbiota in the intestine and the immune system of gestating sows and to improve gut health. Thus, DF may help alleviate the adverse effects of the stressful production cycle of gestating sows. These benefits may subsequently result in improved reproductive performance of sows. Previous studies have reported changes in microbiota by providing gestating sows with DF, and the responses of microbiota varied depending on the source of DF. The responses by providing DF to gestating sows were inconsistent for antioxidative capacity, hormonal response, and inflammatory response among the studies. The effects of DF on reproductive performance were also inconsistent among the previous studies. Potential reasons contributing to these inconsistent results would include variability in reproductive performance data, insufficient replication, influence of other nutrients contained in the DF diets, characteristics of DF, and experimental periods. The present meta-analysis suggests that increasing the total DF concentration by 10 percentage units (e.g., 12% to 22% as-fed basis) in gestating sow diets compared to the control group improves the litter born alive by 0.49 pigs per litter. However, based on the present review, questions remain regarding the benefits of fibers in gestating sow diets. Further research is warranted to clarify the mode of action of fibers and the association with subsequent reproductive performance in gestating sows.

## INTRODUCTION

The life cycle of sows in modern swine industry can be intense and stressful, which may potentially affect their health and performance. The adoption of hyper-prolific sows to increase total litter size and piglet production per sow per year has become widespread in the industry, but this has led to side effects such as constipation, longer farrowing duration, and greater variation in litter birth weight, which may overburden sows [1,2]. Many researchers have observed reproductive performance problems, including reduced litter born alive, in gestating sows due to this stressful life cycle and the side effects associated with hyper-prolificacy [3–5].

Swine nutritionists are currently investigating alternative feed additives and feeding strategies to alleviate the reproductive performance problems without using antibiotics [6–8]. Among the alternative strategies, the addition of dietary fiber (DF) has been widely used as prebiotics in livestock to modulate the gut microbiota and immunological status, which can improve sow health and reproductive performance [9,10]. Previous meta-analyses have shown that the effects of DF on reproductive performance depend on the fiber source, neutral detergent fiber content, and parity, with a significant interaction between these factors and litter size born alive [11]. Updated reviews have suggested that more appropriate measures of fiber are soluble dietary fiber (SDF) and insoluble dietary fiber (IDF), and that the effect of supplemental DF is better when it is provided during one or more reproductive cycles [12]. Despite decades of research, questions remain about the effects of high-DF diets on gestating sows. Therefore, the objectives of the present review were to summarize the current data on the effects of DF on gestating sows and to provide an overview of the application of DF in gestating sow diets.

## JUSTIFICATION FOR ADDING FIBER TO GESTATING SOW DIETS

Prolific sows often experience challenges related to farrowing duration, digestion, and digestive microbiota [1,13]. Prolonged farrowing duration can result in higher numbers of piglet deaths at birth, lower piglet survival rates, greater postpartum oxidative stress, and increased incidence of sow anorexia, ultimately leading to reduced productivity of litter piglets. These challenges can be intensified by factors such as constipation, oxidative stress, and insulin resistance, which can cause inadequate physical endurance during farrowing [5,14,15].

Digestive transit is also commonly affected in late gestation and plays a crucial role in the farrowing process and early lactation. Constipation can lead to dysbiosis and uncontrolled growth of undesired bacteria in the gut, causing digestive discomfort for the sow and potential health issues [16]. Therefore, maintaining a balanced digestive microbiota and optimal digestive function is a critical challenge that must be addressed with prolific sows.

Some prevention strategies include avoiding the abrupt change from high-fiber gestation diets to high energy with low-fiber lactation diets [17], adding the fiber source in a diet around farrowing period to optimize digestive transit [18], and using the prebiotics and probiotics to help balance the digestive microbiota [19].

## POTENTIAL MODE OF ACTION OF DIETARY FIBER

The addition of DF to adult sow diets requires a clear understanding for their mode of action. Some researchers have proposed that the positive effect of adding DF to gestating sow diets on reproductive performance primarily arises from two factors: i) modulation of intestinal microbiota and ii) modulation of physiological status. These factors contribute to adequate physical endurance during farrowing, consequently, improving reproductive performance. Also, some researchers have proposed that positive modulation of microbiota and physiological status of sows can directly affect the fetus.

### Effects on intestinal microbiota

The DF provides an essential fermentative substrate to the microbiome and is known to impact microbial composition, diversity, and metabolic capabilities [20,21]. The addition of DF to gestating sow diets resulted in a clear separation of the microbiota in gut or feces among the treatments (Table 1). It appears that separation of the microbiota depends on the different DF sources. In 10 experiments, 5 out of 19 DF-containing groups had a changed microbial diversity in the gut as compared with the control group. Three experiments reported a significant change in microbial diversity, while others failed to detect significance, which could be attributed to difference in feeding length. The studies that reported significant changes in microbial diversity fed the diet containing DF throughout the gestation period, whereas the other studies fed the diet from the late periods of gestation except one experiment. These findings indicate that a sufficient period for fiber consumption is needed to significantly change the microbial diversity of the gut [22,23].

Microbiota are crucial for maintaining the nutrition status, physiology, and immune function of pigs [24,25]. Major or frequent changes in microbiota are often associated with ill health [26–28]. The *Firmicutes* and *Bacteroidetes* are generally the two dominant phyla, which make up about 90% of the fecal microbiota [29]. The *Firmicutes* bacteria are Gram-positive and play a key role in the nutrition and metabolism of the host through the synthesis of short-chain fatty acids (SCFA). Their metabolic products affect other tissues and organs, regulating hunger and satiety. In contrast, *Bacteroidetes* bacteria are Gram-negative and associated with immunomodulation. Their components, lipopolysaccharides and flagellin, interact with cell receptors and enhance immune reactions through cytokine synthesis [30]. The *Firmicutes-*to -*Bacteroidetes* ratio (F:B) is associated with maintaining homeostasis, and changes in this ratio can lead to various pathologies. For example, increased F:B is associated with the development of obesity, while decreased F:B is associated with the intestinal inflammation [31,32]. However, the interpretation of the F:B can vary depending on the viewpoint. For example, an increased F:B can be interpreted as the development of anti-inflammation, while a decreased F:B can be interpreted as anti-obesity. In 10 experiments, 9 out of 19 DF containing groups had a decreased F:B, while 4 DF containing groups had an increased F:B in the gut compared to the control group. The discrepancy observed among these studies can be attributed to the initial state of the gut microbiome in the sow groups before the start of the experiment. The effect of DF is likely dependent on the pre-existing status of the sow gut microbiota prior to the initiation of the experiment. The groups with a reduced F:B compared to the control group indicate that the sows fed DF deposited less energy under the same calorie intake. This not only prevents sow obesity but also allows undeposited energy to be allocated to the fetus or mammary glands. The prevention of sow obesity reduces farrowing duration [1], and this may be a potential mode of action of DF. Likewise, the groups with an increased F:B indicate that the sows fed DF had an improved ability to fight against inflammation, which may lead to energy deposition in the fetus or mammary gland. Therefore, the use of DF seems to balance the F:B, consequently treating obesity or intestinal inflammation.

At the phylum level, 14 out of 19 DF-containing groups showed a positive change in the relative abundance of microbiota compared to control groups. The increase in beneficial bacteria can enhance intestinal SCFA synthesis, which not only provides an important energy source contributing to gut health of host [33] but also downregulates the synthesis of hunger-suppressing hormones such as leptin, peptide YY, and glucagon-like peptide [34]. The SCFA-producing microorganisms, including *Bacteroides*, *CF231*, *Eubacterium*, *Oscillospira*, *Parabacteroides*, *Prevotella*, and *Ruminococcacea*, were increased. Xu et al [35] reported that sows fed diets supplemented with 2.0% guar gum and pregelatinized waxy maize starch during the gestating period increased the relative abundance of *Eubacterium*, *Oscillospira*, and *Ruminococcacea*. This result is consistent with the finding suggested by Li et al [36], who showed that 1.6% inulin addition increased SCFA-producing microorganisms such as *CF231* and *Prevotella*, as well as reduced endotoxin production and thus reduced the intestinal inflammatory response [18,36]. The decrease in harmful bacteria can reduce potential damage to the gut microbial barrier and prevent endotoxin from permeating the blood [37]. The endotoxin-producing microorganism, including *Cyanobacteria, Deuslfovibrio*, and *Oscillibacter*, were decreased. Lu et al [18] reported that sows fed 2.0% resistant starch or konjaku flour from the day of 85 gestation to farrowing showed a decrease in the relative abundance of *Deuslfovibrio* and *Oscillibacter*. These positive changes in the relative abundance of microbiota may ultimately improve reproductivity by reducing metabolic syndrome. Although almost all DF-containing groups showed a positive change in the relative abundance of microbiota, some groups did not show significant differences compared to the control group. The limited information on the characteristics of DF makes it difficult to explain these contrasting results. Further research is required to verify the effects of the characteristics of DF, such as soluble or insoluble, on the gut microbiota.

### Effects on endocrine and metabolic status

To confirm the effect of DF during gestation on the endocrine and metabolic status of sows, response criteria were classified into 4 categories including hormones, immune status, antioxidant index, and metabolite index (Table 2). Eleven studies out of 18 studies measured hormones related to feed intake, farrowing, lactation, and stress and showed a positive response in 7 studies. Although estrogen, insulin, leptin, lutropin, oxytocin, prolactin, serotonin, and stress hormones showed a positive response, not all studies have reported consistent results. Reproductive hormones, including estrogen, progesterone, and lutropin, play an important role in the regulation of female reproduction. Vallet et al [38] suggested a positive relationship between litter size and plasma estrogen on day 110 of gestation. A surge in lutropin triggers the production of progesterone by the corpus luteum, which contributes to pregnancy maintenance, embryo survival, and placental development and function [39,40]. In addition, Li et al [10] reported an increase in placental weight in sows fed a high-fiber diet over an extended period spanning the second and third parities, which was attributed to alterations in plasma concentrations of reproductive hormones.

Insulin regulates carbohydrates, fat, and protein metabolism by promoting the absorption of glucose from the blood into the liver, fat, and skeletal muscle cells. It has been proposed that greater insulin resistance may be the potential cause of longer than optimal farrowing durations in women [41], as well as greater than optimal production of reactive oxygen species (ROS) due to metabolic disorders. Additionally, Père and Etienne [42] reported that gestating sows become resistant to insulin towards the end of gestation. In a previous study conducted by Xu et al [35], SDF was found to alleviate insulin resistance in perinatal sows by increasing the level of circulating odd-chain fatty acids in plasma. However, while some studies have shown that DF delays postprandial peaks of insulin concentrations compared to sows fed a control diet [43], there have been no significant differences in insulin resistance between sows fed a DF-added diet and a control diet in other studies [44–46].

Leptin is an appetite-suppressing hormone that is secreted after consuming diets and acts on the brain to induce satiety [47]. Sows fed high DF during gestation experienced decreased plasma leptin concentrations before farrowing, which negatively correlated with the feed intake of sows during lactation [43,48]. However, recent research has focused on the effect of long-term DF consumption on serum leptin levels compared to a control diet through a meta-analysis in humans. These findings suggest that consuming DF over an extended period may lower serum leptin levels primarily in obese individuals [49]. A possible reason for increased lactation feed intake in sows fed high DF during gestation is that the increased size and capacity of the digestive tract may facilitate the adaptation of sows to the drastic increase in feeding intake required during lactation [50].

Prolactin is a crucial hormone for initiating and maintaining milk production [51]. Prolactin is involved in the cell proliferation, development of mammary glands, and secretion of milk. This hormone eventually helps provide nutrients to suckling piglets through sow milk and improving survival rates of offspring. High DF intake tended to increase prolactin concentrations in gestating sows [43,52], but other studies showed no effect on prolactin concentrations [44,46]. The potential reasons for these discrepancies among the studies may be associated with maternal obesity during the gestation period. According to the results of Lepe et al [53] who investigated the effect of maternal obesity on lactation, obese mothers had lower prolactin concentrations, which led to delayed lactogenesis. Therefore, preventing obesity in gestating sows through high DF intake may increase prolactin concentration in serum.

Oxytocin is a neurohypophysial hormone that plays a central role in the regulation of farrowing and lactation, such as the initiation of uterine contractions and milk secretion [54]. Li et al [10] reported that sows fed a diet supplemented with 2.26% inulin and 18.2% cellulose during the gestational period had increased plasma oxytocin levels, which were associated with postprandial satiety due to the high-DF diet consumption [55].

Among the hormones related to stress, cortisol in serum is a criterion that reflects stress intensity [56]. Several studies have reported that high-DF diets can influence welfare by decreasing stereotypical behavior that leads to cortisol stimulation [57,58]. A recent study suggested that a diet containing 5% resistant starch during the gestation period contributed to enhancing postprandial satiety, alleviating stress status, and reducing abnormal behaviors [59], but another study reported that stress hormones were not affected in sows fed a diet containing 40% soybean hulls as a fiber source [60]. These inconsistent results were likely due to differences in fiber source and type.

Eight out of 18 studies have measured the criteria of immune status and showed a positive response in 7 studies. Inflammation is a biological response of the immune system to harmful stimuli, including pathogens, damaged cells, toxic compounds, or irradiation [61]. Inflammatory stimuli activate intracellular signaling pathways that then activate the production of inflammatory mediators, including pro-inflammatory factors such as interleukin-6 and tumor necrosis factor-α. These factors have been used as primary criteria to determine the immune status. The studies reported a positive response in immune status, suggesting that sows fed high DF generate more microbiota-derived SCFA that enhances the barrier function in intestinal epithelial cells [18, 48,62]. Enhanced barrier function in the intestine decreases the gut permeability, which leads to the prevention of the inflow of endotoxins. Consequently, high DF fed to sows may reduce systemic inflammation. Another possible reason for the altered immune status is that DF could promote intestinal peristalsis and excretion of stool to reduce the incidence of gastrointestinal disorder such as constipation which may increase the absorption of harmful microbial endotoxins [18].

Six out of 18 studies measured antioxidant markers and showed positive responses in 4 studies. Oxidation is typically initiated by ROS produced by the metabolism of cells. Free radicals or ROS, in general, are known to play both detrimental and beneficial roles [63]. Low ROS levels interact with specific targets and play an essential role in redox signaling involved in stress adaptation, homeostasis, and health maintenance [64]. Conversely, high exposure to ROS affects non-specific targets and induces oxidative stress, such as lipid peroxidation, damaged DNA and cell death, leading to reduced immunity and resistance to various diseases [65]. Complex enzymatic systems containing catalase, superoxide dismutase, and glutathione peroxidase, as well as nonenzymatic systems containing glutathione, beta-carotene, and vitamin E, play vital roles in protecting organisms from oxidative damage [66]. The antioxidant enzymes and oxidative products such as malondialdehyde and protein carbonyl are usually used as biomarkers of oxidative stress. The increased demands of energy and oxygen for the placenta of sows in late gestation lead to excessive oxidative stress [67]. The oxidative stress in gestating sows fed a high-DF diet has been alleviated by increasing antioxidant capacity [68–70]. A study that supplemented gestating sow diets with 0.8%, 1.6%, or 2.4% inulin, resulting in a total dietary fiber (TDF) content of 27%, showed an increase in the antioxidant capacity of gestating sows [46]. This result is consistent with the result of Liu et al [70], who showed that wheat bran, soybean hulls, and sugar beet pulp addition, consequently containing 33% TDF, increased glutathione peroxidase or decreased malondialdehyde. However, another study reported that sows fed diets with the same TDF content of 19% but different IDF- to-SDF ratios (IDF:SDF) of 3.9, 5.6, 9.1, and 12.8 showed different oxidative statuses [71]. This study observed that the antioxidant capacity was improved when the IDF:SDF was less than 5.59, implying that the composition of DF in gestating diets played an important role in improving antioxidant capacity.

Twelve out of 18 studies have measured metabolites to verify energy status and nutrient metabolism, and 9 of these studies showed a positive response. However, the effects of DF on serum metabolites were inconsistent among the studies. Glucose concentration has been used as an indicator of energy status and diabetic tendencies [72], as sows can become glucose intolerant and have diabetic tendencies during pregnancy [73,74]. Supplying high DF to sows can be expected to prevent rapid increases in blood glucose due to delayed postprandial peaks [43]. However, there have been no studies demonstrating the effects of high DF on alleviating glucose tolerance in gestating sows. Because glucose intolerance is more pronounced in obese mothers [75], evaluating the effects of high DF on glucose tolerance in non-obese mothers may not be relevant or applicable to obese mothers, and may therefore lead to misinterpretation. This acknowledges that further research may be necessary to fully understand the effects of high DF on glucose tolerance in obese mothers during pregnancy.

Urea serves as a nitrogen source for gut microbes and can provide an estimate of the state of protein metabolism in pigs. While most studies suggest that feeding sows a high-DF diet does not significantly affect blood urea, some studies show a decrease in blood urea. The fermentation of DF in the gut can influence microbial mass and activity [76,77]. However, highly fermentable fibers tend to increase microbial mass, which results in a greater transfer of urea from the blood to the gut, thereby reducing plasma concentrations, as seen in rat studies [78]. Alternatively, lower plasma urea concentrations may indicate lower protein oxidation in the liver, which may be related to reduced intestinal protein absorption when sows are fed a high-DF diet [44].

It is well known that non-esterified fatty acids (NEFA) are a product of fat metabolism and a good indicator of catabolism of fat reserves [79]. Several studies have reported that feeding high DF to sows reduces NEFA in their serum during late gestation, suggesting that high DF might reduce fat catabolism and thus preserve body reserves [35,62]. Indeed, Shang et al [62] showed that sows fed sugar beet pulp had less body fat loss during lactation, but no significant difference in backfat loss was observed between treatments. In contrast, wheat bran supplementation did not affect serum NEFA concentrations compared to the control diet. Previous studies have shown a negative correlation between SCFA production and serum NEFA concentration, indicating that fermentable fiber can decrease serum NEFA concentration by increasing SCFA production [80]. Sugar beet pulp contains more SDF that is readily fermentable than wheat bran, leading to increased production of SCFA in sows fed sugar beet pulp as indicated by increased fecal concentration of total SCFA. The SCFA are composed of approximately 60% acetic acid, 25% propionic acid, and 15% butyric acid, respectively [81]. Acetic acid may modulate insulin sensitivity by reducing fatty acid flux [82], while butyrate is almost completely used by colonocytes as their preferred energy substrate [83]. Propionate is associated with positive effects on metabolic health, such as lowering serum cholesterol [84]. Therefore, increased total SCFA are beneficial in maintaining blood lipid levels and alleviating the inflammatory state of sows.

The results regarding the effect of high DF fed to sows on endocrine and metabolic status were inconsistent among the studies. The main reasons for the discrepancy in results could be attributed to the characteristics of DF used, the body condition scores of gestating sows during the experiment, and the experimental environment, particularly in high-temperature conditions. Further research is required to verify the interaction between the characteristics of the DF and the body condition scores of gestating sows.

### Effects on reproductive performance

Balanced microbiota and reduced systemic inflammation due to DF may improve reproductive performance in gestating sows. Moreover, a previous meta-analysis of reproductive performance with DF supplementation showed that the number of pigs born alive increased by 0.4 piglets per litter when gestating sows were fed additional DF [11,85]. However, individual studies have reported inconsistent results regarding the effects of DF supplementation in the gestating diet on reproductive performance. Some studies showed no significant effect, while others showed positive or negative results. Similarly, the results of individual studies in the present meta-analysis showed no significant effects of a high-DF diet on any sow performance traits except for lactation feed intake and litter weaning weight (Table 3). One reason for the inconsistent results is the large variation often observed in reproductive data. Large replication per diet is needed to detect effects and to avoid drawing inaccurate conclusions. For instance, to detect a 10% difference with a statistical probability level of p<0.05 in litter size (about 1.0 pig/litter) among diets, at least 63 replications per diet would be required [86]. Another reason for the inconsistent results is that factors other than the elevated cell wall content of fibrous ingredients, such as differences in amino acids, vitamins, and trace mineral content, may also be important [85].

**Figure 1:**
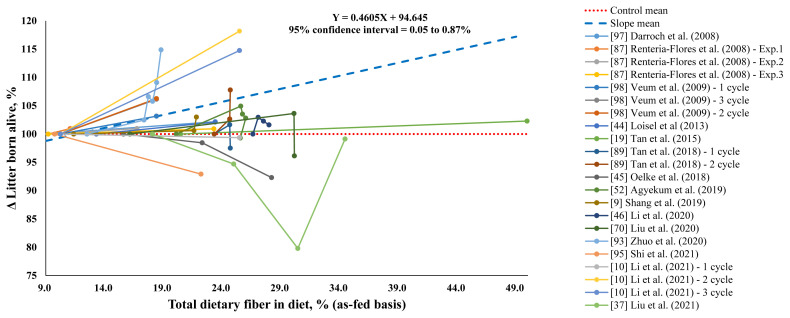
Effects of total dietary fiber (TDF) concentrations on changes (Δ) in litter born alive of the sows fed a fiber-supplemented diet compared with those fed a control diet. The slope mean (blue-colored dashed line) represents the mean of the linear slopes (n = 22). A linear slope was calculated for each experiment, and the slope data were pooled to calculate the mean slope and their 95% confidence interval using the UNIVARIATE procedure of SAS (SAS Inst. Inc., Cary, NC, USA). This linear slope indicates that a 1 percentage unit increase in dietary TDF concentration (as-fed basis) leads to a 0.46% increase in the number of piglets born alive per litter, as per the following equation: Y = 0.4605X+94.658.

In this meta-analysis, we examined 15 published reports dating from 2008 to 2022 that reflect SDF and IDF as characteristic components of DF. For studies that did not provide information on SDF and IDF of ingredients, book or reference values were used to calculate these values. The concentration of TDF, SDF, and IDF in control diets ranged from 8.89% to 26.6%, 1.10% to 5.00%, and 7.30% to 23.0%, respectively, while the range for test diets was 11.1% to 49.9%, 1.40% to 7.50%, and 7.88% to 45.9%, respectively. The weighted averages of TDF, SDF, and IDF concentration in control and test diets according to number of observations were 11.7% and 21.5%, 1.27% and 2.60%, and 7.25% and 18.9%, respectively. The effects of DF addition on reproductive performance were inconsistent among individual studies. However, in this meta-analysis, a 1 percentage point increase in TDF concentration (as-fed basis) compared to the control group improved the litter born alive by 0.46% (95% confidence interval = 0.05% to 0.87%; Figure 1). Based on the weighted average of TDF concentrations and litter born alive in both the control diets (weighted average of TDF = 11.7%) and test diets (weighted average of TDF = 21.5%), a 10 percentage unit increase in TDF concentration compared to the control group improved the litter born alive by 0.49 pig per litter, which is in agreement with a previous meta-analysis [12].

The type of DF used in gestation diets may contribute to the discrepancies observed among studies. For instance, sows fed a high-SDF diet during gestation had higher numbers of live embryos and total embryo survival rates compared to sows fed a high-IDF diet [87]. However, Liu et al [70] showed that sows fed high-SDF or -IDF diets in the last gestation period could improve maternal immune function and redox status. Nonetheless, a high maternal SDF intake increased pre-weaning mortality and decreased the number of weaned piglets compared to sows fed an IDF diet. A recent study also revealed that IDF and SDF content, as well as the IDF:SDF, varied greatly among different fiber resources, leading to dramatic changes in fermentation kinetics parameters of gas production [88]. The IDF:SDF in a fiber resource could affect overall diet utilization and play an important role in improving the reproductive performance of sows [87]. In addition, recent studies found that the IDF:SDF had a significant effect on the health status of sows and their offsprings. Higher average piglet body weight and litter weight at weaning were observed when the IDF:SDF was 3.89 in the gestation diet [46,71]. Furthermore, a study using purified inulin and cellulose in gestation diets has been conducted to avoid the effect of fiber composition on reproductive performance [10]. This study investigated the effects of fiber addition with the same IDF:SDF and different TDF concentrations on sow and litter performance and reported that TDF addition to the gestation diet with an equal IDF:SDF during gestation promoted the physical status of sows and improved sow and litter performance. Some studies have reported that high-SDF diets can help improve reproductive performance by producing more SCFA [9,87,89]. However, it was challenging to distinguish the effect of a specific fiber type on sow reproductive performance through this meta-analysis. Recently, a study investigated the possible mechanism of DF improving sow reproductive performance [90]. They prepared two experimental diets: a semi-purified basal diet (non-fiber diet) and a fiber diet, which was the basal diet supplemented with 0.83% inulin and 20% cellulose. Then, they investigated fetal growth and placental development and function and reported that DF supplementation during gestation could increase maternal serum serotonin levels by promoting colonic serotonin synthesis, in which gut microbiota might be involved. In addition, DF supplementation during gestation promoted the transport of serotonin from the mother to the placenta in sows, improved placental development and function, and ultimately promoted fetal growth.

Sows that were fed high-DF diets for multiple reproductive cycles have shown greater benefits from the feeding of high-DF diets during gestation. In a previous meta-analysis, sows fed high DF during gestation in multiple-cycle studies produced 0.5 more pigs at weaning than those fed the control diet. However, in studies involving only one reproductive cycle, sows fed high DF produced 0.2 fewer pig at weaning than sows fed the control diet [12]. In this review, three out of 15 studies were conducted for multiple reproductive cycles and reported an improvement in litter born alive or the number piglet at weaning. A recent study reported that total born and born alive were similar among all treatments in the first parity, but they were greater for sows fed diets containing a 15%-point unit higher TDF content than the control diet in the second and third parity [10]. Unlike one-cycle studies, multiple-cycle studies have consistently shown that the addition of DF during gestation leads to an improvement in litter born alive or the number piglet at weaning. These results suggest that feeding long-term DF is required to modulate the microbiota in the intestine and reduce systemic inflammation, which may result in significant differences in reproductive performance.

Ten out of 15 studies reported that feeding DF during gestation increased feed intake in the lactating period. There are two potential reasons for the increase in feed intake. Firstly, the gestation diet containing high DF is more fermentable than the control diet, which can improve insulin sensitivity and increase feed intake [9,89]. Secondly, feeding a high-DF diet during gestation can increase the bulkiness of the diet, helping sows adapt to the sudden increase in feed intake required to meet the demands of lactation. [52,91]. The increased lactation feed intake can lead to increased milk production, resulting in a greater growth rate of piglets from sows fed high DF.

## CONCLUSION

The effect of high-DF diets on intestinal microbiota, endocrine and metabolic status, as well as reproductive performance in gestating sows, remains inconsistent among individual studies. However, meta-analysis has demonstrated positive results, particularly in terms of litter born alive, indicating consistent improvement in reproductive performance when high-fiber diets are fed for multiple reproductive cycles. Based on this review, feeding gestating sows with diets containing around 21% TDF can be expected to increase litter born alive by 0.5 pigs per litter, and long-term feeding can further enhance the positive effects of high fiber diet. Nonetheless, the precise mechanisms underlying the benefits of DF in gestating sows remain unclear. Further research is needed to elucidate the mechanisms of action of DF and its association with subsequent reproductive performance in gestating sows.

## Figures and Tables

**Table 1:** The effects of dietary fiber on the fecal and gut microbiota of gestating sows^1)^

Main source	Inclusion rate (%)	Fiber composition in diet^2)^ (%)				Feed allowance^3)^ (kg/d)		Feeding length	Microbial diversity	Changing relative abundance			References
		
		Control		Treatment		Control	Treatment			Phylum level		Another taxon	
		
		SDF	IDF	SDF	IDF								
Konjac flour	2.20	2.30	18.16	4.00	45.93	2.42	2.41	Gestating period	-	-	-	○	[19]^5)^
Guar gum plus Pregelatinized waxy maize starch	2.00	Not provided		Not provided		2.42		Gestating period	↑	○	F:B^4)^(↑)	○	[35]
Stevia residue	20.0	Not provided		Not provided		2.76	3.70	Gestating period	↑	○	F:B (↑)	○	[92]^6)^
	30.0												
	40.0												
Wheat bran	18.0	1.57	10.91	2.02	15.33	2.40	2.56	Gestating period	×	○	F:B (↓)	○	[93]^6)^
Wheat bran and fiber mix	16.4 / 1.0			2.16	15.56								
(Wheat bran / fiber)	14.8 / 2.0			2.29	15.79								
	13.2 / 3.0			2.42	16.01								
	11.6 / 4.0			2.56	16.24								
Inulin	1.60	Not provided		Not provided		3.30		d 80 of gestation to farrowing	×	○	F:B (↓)	○	[36]
Alfalfa meal	10.0	2.12	15.9	2.23	22.74	Recommendations of NRC (2012)		d 60 of gestation to farrowing	×	○	×	○	[37]^5)^
Wheat bran	30.0	1.39	9.98	1.86	19.95	3.00	3.31	d 80 of gestation to weaning	×	○	×	○	[62]^6),7)^
Sugar beet pulp	20.0			4.06	17.54		3.07				×		
Fine wheat bran	20.0	2.20	18.28	2.79	17.55	2.51		Gestating period	↑	○	F:B (↓)	○	[48]
Inulin	0.83	0	0	0.83	20.00	2.40	2.90	Insemination to d 106 of gestation	×	○	F:B (↓)	○	[90]
Cellulose	20.0												
Lignocellulose	1.50	2.18	17.31	2.10	18.00	3.00	3.03	d 85 of gestation to farrowing	×	○	×	○	[18]^5),6)^
Resistant starch	2.00			4.10	16.50		3.04				×		
Konjac flour	2.00			2.17	14.84		3.04				F:B (↓)		

1) A circle sign (○) represents significant difference at p<0.05, a multiplication sign (×) represents no difference at p>0.05, an up-arrow sign (↑) and down arrow sign (↓) represents significant increase and decrease at p<0.05, respectively.

2) SDF, soluble dietary fiber; IDF, insoluble dietary fiber; IDF:SDF, insoluble dietary fiber to soluble dietary fiber ratio.

3) Weighted average value based on feeding length during gestating period.

4) F:B, Firmicutes to Bacteroidetes ratio.

5) The fiber composition in diet was calculated value based on previous study [9,12,19,76].

6) The control and treatment groups were adjusted to have similar metabolizable or digestible energy intake through different feed intake.

7) Dietary fiber composition of lactation diet; the control, wheat bran, and sugar beet pulp diets contained 11.8%, 16.8%, and 16.9% total dietary fiber, 1.43%, 2.72%, and 1.70% soluble dietary fiber, and 10.4%, 14.1%, and 15.2% insoluble dietary fiber, respectively.

**Table 2:** The effects of dietary fiber on endocrine and metabolic status of gestating sows^1)^

Main source	Inclusion rate (%)	Fiber composition in diet^2)^ (%)				Feed allowance^3)^ (kg/d)		Feeding length	The presence or absence of a positive response				References
	
		Control		Treatment		Control	Treatment						
		
		SDF	IDF	SDF	IDF				Hormone	Immune	Antioxidant	Metabolite	
Sunflower meal	9.75	Not provided		Not provided		2.40	2.80	Gestating period	○	-	-	×	[43]^4),5)^
Wheat bran	9.75												
Sugar beet pulp	19.50												
Soybean hulls	9.75												
Corn gluten feed	3.00												
Soybean hulls	8.00	1.90	11.4	2.80	20.6	Based on backfat thickness and body weight		d 90 of gestation to farrowing	×	-	-	○	[44]
Wheat bran	8.00												
Sunflower meal	8.00												
Sugar beet pulp	8.00												
Soybean hulls	12.2	5.00	10.6	5.40	16.9	2.26	2.36	d 73 of gestation to farrowing	×	-	-	×	[45]^5)^
	24.4			7.50	20.7		2.56						
Oat straws	10.0	2.70	17.4	2.55	23.0	2.40	2.40	d 86 of gestation to farrowing	○	-	-	×	[52]
				2.50	23.2								
Wheat straws				2.60	23.4				×	-	-	○	
				2.50	23.0								
Inulin	2.50	No control diet		3.87	15.0	No control diet	2.44	Gestating period	-	○	○	-	[71]
Cellulose	0.00												
Inulin	1.50			2.87	16.0								
Cellulose	1.00												
Inulin	0.50			1.87	17.0				-	×	×	-	
Cellulose	2.00												
Inulin	0.00			1.37	17.5								
Cellulose	2.50												
Resistant starch	5.00	2.23	16.2	7.28	17.6	2.57		Gestating period	○	-	○	-	[69]^4)^
Fermented soybean fiber	5.00			Not provided					×	-	○	-	
Inulin	0.80	3.63	23.0	4.34	22.7	3.30		d 80 of gestation to farrowing	×	-	○	-	[46]
	1.60			5.05	22.5				×	-	○	-	
	2.40			5.77	22.2				×	-	○	-	
Wheat bran	16.4	1.92	15.6	3.22	29.7	3.00	3.20	d 90 of gestation to farrowing	-	○	○	×	[70]^5)^
Soybean hull	16.4												
Wheat bran	12.0			5.06	28.4				-	○	○	×	
Soybean hull	12.0												
Sugar beet pulp	11.4												
Wheat bran	20.0	No control diet		Not provided		No control diet	2.12	d 30 of gestation to weaning	-	-	-	×	[94]^4)^
Soya hulls	20.0						2.15		-	-	-	○	
Rice hulls	20.0						2.20		-	-	-	×	
Guar gum plus pregelatinized waxy maize starch	2.00	Not provided		Not provided		2.42		Gestating period	-	○	-	○	[35]
Wheat bran	18.0	1.57	10.9	2.02	15.3	2.40	2.56	Gestating period	-	×	-	○	[93]^5)^
Wheat bran and fiber mix (Wheat bran / fiber)	16.4 / 1.0			2.16	15.6								
	14.8 / 2.0			2.29	15.8								
	13.2 / 3.0			2.42	16.0								
	11.6 / 4.0			2.56	16.2								
Inulin	2.60	1.10	9.14	2.77	30.3	2.27	2.73	Gestating period	○	-	-	○	[10]
Cellulose	18.2												
Alfalfa meal	10.0	2.12	15.9	2.23	22.7	Recommendations of NRC (2012)		d 60 of gestation to farrowing	-	○	○	-	[37]^4)^
Wheat bran	30.0	1.39	9.98	1.86	20.0	3.00	3.31	d 80 of gestation to weaning	-	○	-	×	[62]^5),6)^
Sugar beet pulp	20.0			4.06	17.5		3.07		-	○	-	○	
Wheat bran	36.4	1.58	8.17	2.4	19.7	2.38		Gestating period	×	-	×	×	[95]^4)^
Chicory meal	23.6			Not provided				×	-	×	○		
Soybean curd residue	17.8							×	-	×	×		
Corn gluten	27.0							×	-	×	○		
Rice bran meal	46.5							×	-	×	×		
Fine wheat bran	20.0	2.20	18.3	2.79	17.6		2.51	Gestating period	○	○	-	○	[48]
Inulin	0.83	0	0	0.83	20.0	2.40	2.90	Insemination to d 106 of gestation	○	-	-	-	[90]
Cellulose	20.0												
Lignocellulose	1.50	2.18	17.3	2.10	18.0	3.00	3.03	d 85 of gestation to farrowing	×	○	-	-	[18]^4),5)^
Resistant starch	2.00			4.10	16.5		3.04		○	○	-	-	
Konjac flour	2.00			2.17	14.8		3.04						

1) A circle sign (○) represents significant difference at p<0.05, a multiplication sign (×) represents no difference at p>0.05, no control diet; comparison between treatments.

2) SDF, soluble dietary fiber; IDF, insoluble dietary fiber; IDF:SDF, insoluble dietary fiber to soluble dietary fiber ratio.

3) Weighted average value based on feeding length during gestating period.

4) The fiber composition in diet was calculated value based on previous study [9,12,19,76,96].

5) The control and treatment groups were adjusted to have similar metabolizable or digestible energy intake through different feed intake.

6) Dietary fiber composition of lactation diet; the control, wheat bran, and sugar beet pulp diets contained 11.8%, 16.8%, and 16.9% total dietary fiber, 1.43%, 2.72%, and 1.70% soluble dietary fiber, and 10.4%, 14.1%, and 15.2% insoluble dietary fiber, respectively.

**Table 3:** The effects of dietary fiber on reproductive performance of gestating sows^1)^

Main source	n	Fiber composition in diet^2)^ (%)						Feeding period	Δ Litter size^3)^, No./Litter				Δ Litter weight^3)^ (kg)		Δ FI^3),4)^ (kg/d in lactation)	References

		Control			Treatment											
			
		TDF	SDF	IDF	TDF	SDF	IDF		Total born	Still born	Born alive	Weaned	At birth	Weaned		
Soybean hulls	64	8.89	1.59	7.30	23.41	2.95	20.45	Gestating period	0.00	-	0.22	0.48	−0.65	0.66	0.24	[97]^5)^
Oat bran	124	9.21	1.55	7.66	11.07	3.19	7.88	Gestating period	−0.20	-	0.10	−0.10	−0.30	−0.49	−0.20	[87]
Wheat straw	119				16.75	1.40	15.35		0.10	-	0.10	−0.20	0.15	−1.12	0.00	
Soybean hulls	131				23.29	3.00	20.29		−0.30	-	0.10	0.00	−0.42	−0.99	0.50*	
Wheat straw	162	1.60	8.62	5.39	18.41	1.45	16.95	Gestating period^6)^ (3 reproductive cycle)	-	−0.05	0.31*	0.25	-	1.48	0.34*	[98]^5)^
	111								-	−0.13	0.61*	0.92	-	8.48*	0.35*	
	86								-	−0.10	0.63*	1.01	-	0.84	0.35*	
Soybean hulls	15	13.3	1.90	11.4	23.44	2.80	20.60	d 90 of gestation to farrowing	0.10	-	0.30	0.50	0.50	1.10	0.10	[44]
Konjac flour and Wheat bran	28	20.5	2.30	18.2	49.93	4.00	45.93	Gestating period	0.25	-	0.26	0.18	0.66	6.12*	-	[19]^5)^
Konjac flour	23	23.3	2.51	20.8	24.64	3.78	20.86	Gestating period ^6)^ (2 reproductive cycle)	0.40	-	0.20	0.30	0.10	0.50	0.30*	[89]^5)^
Sugar beet pulp	23				24.69	3.70	20.99		0.20	-	−0.30	0.20	−0.70	0.9	0.10	
Konjac flour	23				24.64	3.78	20.86		−0.20	-	0.30	1.00*	0.30	13.0*	0.90*	
Sugar beet pulp	23				24.69	3.70	20.99		0.30	-	0.88	0.40	0.60	3.40	0.20	
Rice bran Soybean hulls	11	15.6	5.00	10.6	22.30	5.40	16.90	d 73 of gestation to farrowing	−0.50	−0.40	−1.00	-	−1.40	3.50*	0.20	[45]
	11				28.20	7.50	20.70		−1.20	−0.40	−0.20	-	−0.30	3.80*	0.00	
Oat straw	30	20.1	2.70	17.4	25.58	2.55	23.03	d 86 of gestation to farrowing	-	−0.39	0.70	0.50	-	5.26*	0.45*	[52]
	30				25.73	2.50	23.23		-	−0.83	0.50	0.70	-	10.9*	0.45*	
Wheat straw	30				26.00	2.60	23.40		-	−0.15	0.40	0.50	-	3.31	−0.13	
	30				25.54	2.50	23.04		-	−0.70	−0.10	0.40	-	2.26	0.11	
Wheat bran	15	11.4	1.39	9.98	21.81	1.86	19.95	d 107 gestating to weaning	−0.07	−0.40	0.37	0.54	0.76	5.74	0.36*	[9]^5),8)^
Sugar beet pulp	15				21.60	4.06	17.54		−0.20	−0.26	0.10	0.34	0.42	7.45*	0.68*	
Inulin	22	26.6	3.63	23.0	27.06	4.34	22.72	d 80 of gestation to farrowing	0.23	0.40	0.40	0.41	1.55	10.94*	1.084*	[46]
	22				27.51	5.05	22.46		−0.36	0.31	0.28	0.71	3.25	14.68*	1.238*	
	22				27.98	5.77	22.21		−0.05	0.22	0.22	0.82*	0.70	5.08	0.562	
Wheat bran	20	17.5	1.92	15.6	32.90	3.22	29.68	d 90 of gestation to farrowing	0.82	0.06	0.5	0.66	1.11	3.86	−0.19	[70]^5)^
	20				33.50	5.06	28.44		0.08	0.47	−0.53	−0.67	0.22	−8.35	−0.15	
Wheat bran and fiber mix	35	12.5	1.57	10.9	17.35	2.02	15.33	Gestating period	0.80	-	0.30	0.20	0.10	0.20	0.33	[93]
	35				17.72	2.16	15.56		0.90	-	0.80	0.40	0.90	3.7	0.50*	
	35				18.08	2.29	15.79		1.20	-	0.70	−0.20	1.20	2.2	0.59*	
	35				18.43	2.42	16.01		1.00	-	1.10	−0.20	1.70	1.2	0.26	
	35				18.80	2.56	16.24		1.90	-	1.80	−0.20	1.90	−0.9	0.46*	
Wheat bran	12	9.75	1.58	8.17	22.15	2.40	19.75	Gestating period	0.54	-	−0.79	−0.37	-	-	-	[95]^5)^
Inulin and Cellulose	14	10.24	1.10	9.14	25.47	2.77	22.70	Gestating period^6)^ (3 reproductive cycle)	−0.02	-	−0.07	−0.53	0.62	−1.61	0.55*	[10]
	13								2.11*	-	2.00*	0.27*	4.11*	7.38*	0.51*	
	11								1.10*	-	1.80*	0.55*	3.39*	13.9*	0.31*	
Alfalfa meal	16	18.06	2.12	15.94	24.98	2.23	22.74	d 60 of gestation to farrowing	−0.98	-	−0.67	−0.02	−0.32	6.40	1.01*	[37]
Sugar beet pulp	16				30.43	2.54	27.90		−2.67	-	−2.56	−0.01	−2.8	3.48	0.05	
Soybean hulls	16				34.15	2.59	31.86		0.00	-	−0.11	−0.01	0.91	−0.23	−0.09	
Maximum		26.6	5.00	23.0	49.9	7.50	45.9		2.11	0.47	2.00	1.01	4.11	14.7	1.24	
Minimum		8.89	1.10	7.30	11.1	1.40	7.88		−2.67	−0.83	−2.56	−0.67	−2.80	−8.35	−0.20	
Weighted average^7)^		11.6	1.27	7.25	21.5	2.60	18.9		0.10	−0.05	0.31	0.27	0.22	2.49	0.29	

1) Asterisk sign (*) represent the significant difference at p<0.05.

2) TDF, total dietary fiber; SDF, soluble dietary fiber; IDF, insoluble dietary fiber.

3) The increase or decrease in litter size, litter weight and feed intake measured in dietary fiber groups relative to the control group.

4) FI, feed intake.

5) The fiber composition in diet is calculated value based on previous study [9,12,19,52,76,96].

6) Each row represents reproductive performance of each reproductive cycle.

7) Weighted average according to replications per treatments.

8) Dietary fiber composition of lactation diet; the control, wheat bran, and sugar beet pulp diets contained 11.8%, 16.8%, and 16.9% total dietary fiber, 1.43%, 2.72%, and 1.70% soluble dietary fiber, and 10.4%, 14.1%, and 15.2% insoluble dietary fiber, respectively.
